# Dual Role of Mechanisms Involved in Resistance to Predation by Protozoa and Virulence to Humans

**DOI:** 10.3389/fmicb.2018.01017

**Published:** 2018-05-17

**Authors:** Shuyang Sun, Parisa Noorian, Diane McDougald

**Affiliations:** ^1^ithree Institute, University of Technology Sydney, Sydney, NSW, Australia; ^2^School of Biotechnology and Biomolecular Sciences, The University of New South Wales, Sydney, NSW, Australia; ^3^Singapore Centre for Environmental Life Sciences Engineering, Nanyang Technological University, Singapore, Singapore

**Keywords:** pathogenicity, predation, *Vibrio*, evolution, virulence factors, coincidental evolution, protozoa

## Abstract

Most opportunistic pathogens transit in the environment between hosts and the environment plays a significant role in the evolution of protective traits. The coincidental evolution hypothesis suggests that virulence factors arose as a response to other selective pressures rather for virulence *per se*. This idea is strongly supported by the elucidation of bacterial-protozoal interactions. In response to protozoan predation, bacteria have evolved various defensive mechanisms which may also function as virulence factors. In this review, we summarize the dual role of factors involved in both grazing resistance and human pathogenesis, and compare the traits using model intracellular and extracellular pathogens. Intracellular pathogens rely on active invasion, blocking of the phagosome and lysosome fusion and resistance to phagocytic digestion to successfully invade host cells. In contrast, extracellular pathogens utilize toxin secretion and biofilm formation to avoid internalization by phagocytes. The complexity and diversity of bacterial virulence factors whose evolution is driven by protozoan predation, highlights the importance of protozoa in evolution of opportunistic pathogens.

## Introduction

Many bacterial pathogens are able to survive in non-clinical environments, where environmental conditions play an important role in the persistence and infectivity of those bacteria. In fact, most opportunistic pathogens are not transmitted host to host but rather transit in the environment between hosts for significant amounts of time. Thus, the environment impacts not only their survival but also potential infectivity. For example, global warming has been suggested to be responsible for increased outbreaks of disease caused by *Vibrio* spp., as rising sea surface temperatures promote their growth and virulence in aquatic environments (Vezzulli et al., 2013, 2015). The environmental persistence of bacterial pathogens also depends on their ability to adapt to various stresses. Predation by bacterivorous protists is one of the key mortality factors faced by bacteria in the environment. In response, bacteria have evolved many anti-protozoal mechanisms, such as formation of filaments, increases in swimming speed, surface masking, toxin production and biofilm formation, etc. (Matz and Kjelleberg, 2005). Some mechanisms providing resistance to protozoan grazing may also provide advantages during infection of human and animal hosts. As proposed in the review by Erken et al. (2013), protozoan grazing is “a factor driving the evolution of human pathogens in the environment.” This review summarizes the mechanisms of bacterial pathogens involved in both resistance to predation and human pathogenesis, with an emphasis on advances made in the past 5 years. We compare these mechanisms using examples of intracellular and extracellular pathogens.

## Predation by Heterotrophic Protists Impacts Pathogens in Environment

Protists, or protozoa, are single-celled eukaryotes that range in size from 2 to 2000 μm. Protozoa are grouped based on morphology and mechanisms of feeding and locomotion into three groups, flagellates, ciliates, and amoebae. Heterotrophic flagellates and ciliates feed by sweeping food particles into a mouth-like cytostome, while amoebae engulf food particles using pseudopodia. After ingestion, food vacuoles (phagosomes) are trafficked through the phagocytic pathway and subsequently fuse with lysosomes (phagolysosome) enabling digestion of food particles (Fenchel, 1987).

Predation by bacterivorous protozoa is a well-known mechanism for top down control of bacterial communities due to their high feeding rates (Sherr and Sherr, 2002) and their ability to predate on surface-associated bacterial biofilms (Parry, 2004). However, there are also studies demonstrating that many bacteria, including pathogenic species, benefit from interactions with protozoa. Protozoa have been called the “Trojan horses of the microbial world” for their ability to promote the survival of pathogenic bacteria in the environment (Barker and Brown, 1994). For example, pathogenic bacteria that have been shown to have increased survival in the environment after interactions with protozoa include *Campylobacter jejuni* (Trigui et al., 2016; Reyes-Batlle et al., 2017), *Francisella tularensis* (Buse et al., 2017), *Listeria monocytogenes* (Fieseler et al., 2014), *Legionella pneumophila* (Cervero-Arago et al., 2014, 2015), *Mycobacterium leprae* (Wheat et al., 2014), *Stenotrophomonas maltophilia* (Cateau et al., 2014), and *Yersinia enterocolitica* (Lambrecht et al., 2013). Although the mechanisms of increased bacterial survival are not fully understood, it has been suggested that bacteria can obtain nutrients from protozoa. Furthermore, protozoa are not only ubiquitous in the environment, but are also members of gut microbiota in many organisms. The interactions between protozoa and other gut microorganisms can be beneficial or harmful to host health (Burgess et al., 2017; Chabe et al., 2017).

In addition, some protozoal species are able to form dormant cysts that are resistant to a variety of stresses, and these cysts provide encased bacteria a protective niche against adverse conditions. For example, *Escherichia coli, L. monocytogenes, Salmonella enterica*, and *Y. enterocolitica* demonstrated increased resistance to antibiotics and low pH when in *Acanthamoeba castellanii* cysts (Lambrecht et al., 2015). *L. pneumophila* within cysts of *Acanthamoeba polyphaga* was also reported to be resistant to chlorine treatment (Kilvington and Price, 1990). Therefore, protozoa and cysts generated by protozoa can be vectors for pathogenic bacteria and facilitate disease transmission (Denoncourt et al., 2014; Balczun and Scheid, 2017).

In some cases, interactions of bacteria with protozoa induce the expression of virulence traits, and thus, environmental amoebae have been referred to as “training grounds” for bacteria virulence (Molmeret et al., 2005). This is due to the fact that the bactericidal mechanisms used by amoeba and human immune cells, such as macrophage, are conserved. This includes the cell biology of phagocytosis and phagosome maturation. The mechanisms used by macrophage and amoeba to kill engulfed bacterial cells are similar, including H^+^–ATPase related acidification of the phagosome, oxidative burst from reactive oxygen and nitrogen species, use of metal transporters for iron and manganese efflux and copper and zinc influx, nutrient deprivation and the battery of antimicrobial proteins and lysosomal hydrolases expressed (Siddiqui and Khan, 2012; German et al., 2013). Many bacteria have evolved anti-digestion mechanisms that allow them to survive inside of protozoa, and some of these mechanisms also contribute to their virulence during infection of human and animal hosts (Gong et al., 2016; Paquet and Charette, 2016). Consequently, intracellular pathogens that inhabit phagosomal compartments interfere with their maturation and/or are resistant to host killing mechanisms. Thus, bacteria that have evolved to escape the bactericidal mechanisms of amoeba will be better protected (or more virulent) when encountering immune cells. Protozoan grazing has been shown to shape phenotypic and genotypic composition of bacteria community (Jurgens and Matz, 2002). Although there is limited experimental data showing that long-term protozoan grazing results in genotypic changes, it has been reported that protozoa can induce the expression of bacterial virulence in the short term, probably due mainly to changes in gene expression. For example, *Mycobacterium ulcerans* when co-cultured with *A. polyphaga* infected the footpads of mice much faster than the *M. ulcerans* only controls (Azumah et al., 2017). *S. enterica* serotype Typhimurium was shown to enter a hyperinvasive state after passage through *A. castellanii* (Carlson et al., 2007), and intracellular growth in *A. castellanii* also induced the invasion and virulence of *L. pneumophila* in mouse infection models (Cirillo et al., 1999).

The micronutrients, Fe, Mn, Zn, and Cu play important roles in the innate immune system and in antimicrobial activity of macrophages in defense against invading microbial pathogens (Hood and Skaar, 2012; Samanovic et al., 2012). Both protozoa and macrophage use the toxicity of Cu and Zn to kill bacteria in mature phagosomes. Some bacteria are able to avoid damage induced by these metals by the use of efflux pumps. Thus, copper resistance increases not only the environmental survival of bacterial pathogens, but also contributes to virulence *in vivo* (German et al., 2013). *E. coli* strains with mutations in Cu(I)-translocating P1B-type ATPase (*copA*), iron uptake transporters (*feoAB* and *entC*) and manganese uptake transporters (*mntH*), as well as *Pseudomonas aeruginosa* mutants in Cu(I)-translocating P1B-type ATPase (*cueA*) showed reduced grazing resistance against *Dictyostelium discoideum* (Hao et al., 2016). Efflux systems are also involved in antibiotic resistance. For example, the *C. jejuni* RND-type efflux pump, CmeABC which is associated with multidrug resistance, may also be involved in virulence as well as survival in *A. polyphaga* (Vieira et al., 2017). These data highlight the role of these “virulence factors” in persistence and survival in the environment and likely evolved to protect bacteria from predation rather than for protection against antibiotics, given the long history of co-evolution of protozoa and bacteria.

## Protozoan Grazing Promotes Horizontal Gene Transfer

Horizontal gene transfer (HGT) contributes to bacterial adaptation and evolution. For bacterial pathogens, HGT is involved in resistance to antibiotics and virulence during infection (Juhas, 2015) and protozoan grazing has been shown to play an important role in HGT. Due to the indiscriminate feeding of many protozoa, it is likely that there will be a mix of different bacteria encased in a single food vacuole. This would facilitate the interactions of these bacteria in an enclosed system. For example, *E. coli* strains engulfed by the ciliate, *Tetrahymena pyriformis*, exhibited increased rates of conjugation (Schlimme et al., 1997). After a full digestion cycle, the frequency of conjugation increased 2000- to 4000-fold. *Tetrahymena thermophila* has also been reported to cause an increase in HGT by accumulating phage and susceptible bacteria in the phagocytic vacuoles (Aijaz and Koudelka, 2017). Prophages have been shown to be induced after ingestion due to exposure of the bacterium to oxidative stress in the phagosome, resulting in a switch to the lytic cycle and release of free phage particles. The frequency of lysogen formation was shown to increase sixfold as a consequence of being encased within the phagosome. In both cases, the accumulation of bacteria, phage and DNA in protozoan food vacuoles is proposed to be responsible for the increase in HGT. Protozoan grazing also stimulates biofilm formation by grazing resistant bacteria (Matz et al., 2004, 2005) and it has been proposed that the high cell density in bacterial biofilms is also responsible for increased HGT (Madsen et al., 2012).

## Intracellular Pathogens

**FIGURE 1:**
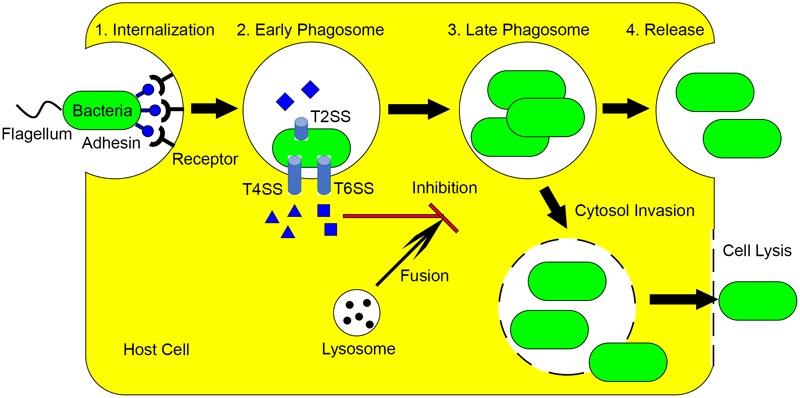
Mechanisms utilized by intracellular pathogens to avoid killing by macrophage and protozoa. (1) Intracellular pathogens utilize flagella and adhesins to invade host cells. (2) After internalization, various effectors are produced through multiple secretion systems, which inhibits the lysosome phagosome fusion. (3) Depending on nutrient conditions, bacteria replicate in phagosome and/or enter cytosol. (4) The intracellular pathogens are released by exocytosis or lysis of host cells.

*L. pneumophila* is the causative agent of Legionnaires’ disease and is one of the best-studied intracellular pathogens. In the environment, *L. pneumophila* interacts with diverse protozoan hosts, which is critical for its persistence as this organism is not transmitted from person to person (Boamah et al., 2017). This further highlights that environmental protozoa are the true hosts for this pathogen. The *L. pneumophila* life cycle within amoebae and macrophage has been described in detail in numerous reviews (Escoll et al., 2013; Richards et al., 2013; Hoffmann et al., 2014). Briefly, *L. pneumophila* (1) invades the host cell after phagocytosis, forming *Legionella*-containing vacuoles (LCVs), (2) uses the Dot/Icm type IV secretion system (T4SS) to inject approximately 300 effector proteins into the host cell, (3) differentiates into the replicative form and proliferates when nutrients are present, (4) when nutrients become limiting, *L. pneumophila* differentiates into the mature infectious form and enters the cytosol, (5) cells are finally released as free swimming cells after host cell lysis (**Figure 1**). Recently, it has been proposed that the *L. pneumophila* life cycle contains even more developmental stages and cell forms (Robertson et al., 2014).

Host cell invasion by *L. pneumophila* is mainly mediated by surface factors like the flagellum and pilus. *L. pneumophila* has a single monopolar flagellum composed of one major subunit, FlaA. A mutation of FlaA reduces the efficiency of invasion of *A. castellanii* and macrophage due to reduced motility (Dietrich et al., 2001; Schell et al., 2016a). In addition, PilY1 is responsible for the biogenesis of the type IV pili, which is important for twitching motility, surface attachment and host cell invasion (Hoppe et al., 2017). PilY1 is also involved in inhibition of fusion of the lysosome with the phagosome in the social amoebae *D. discoideum* and a mutant of PilY1 demonstrated reduced intracellular growth in macrophage (Shevchuk et al., 2014). The mechanisms facilitating host cell invasion are common in other intracellular pathogens. For example, a pathogenicity island in *Mycobacterium avium* encodes proteins inducing actin polymerization of host cells and mutants of this pathogenicity island are deficient in invasion of both macrophage and *A. castellanii* (Danelishvili et al., 2007).

After internalization, the intracellular survival of *L. pneumophila* largely depends on effectors secreted by the T4SS that have similar functions in amoebae and macrophage, probably due to the high similarity of mechanisms of phagocytic trafficking expressed by these two host cells. Most of the effectors work in a redundant manner to block phagosome-lysosome fusion and promote *L. pneumophila* intracellular survival. This has been summarized in earlier reviews (Finsel and Hilbi, 2015; So et al., 2015). Recently, one of the effectors, a cell membrane-localized iron transporter, IroT, was reported to be involved in ferrous iron acquisition and the mutant is defective in intracellular growth in *A. castellanii* and macrophage (Portier et al., 2015). Iron-dependent virulence is widely distributed among bacterial pathogens (Reinhart and Oglesby-Sherrouse, 2016; Butt and Thomas, 2017; Ramakrishnan, 2017). Although there are limited evidences showing that iron-regulated virulence factors contribute to grazing resistance, it has been reported that *E. coli* strains encoding genes for iron uptake survived better with *D. discoideum* than avirulent strains lacking these genes (Adiba et al., 2010). A recent report demonstrates that *E. coli* strains with mutations in iron uptake genes are attenuated for intracellular survival in *D. discoideum* (Hao et al., 2016). Another *L. pneumophila* T4SS effector, RidL, binds with the retromer (protein complex responsible for recycling transmembrane receptors from endosomes to the *trans*-Golgi network) of host cells and inhibits retrograde trafficking, resulting in better intracellular growth in both *D. discoideum* and macrophage (Finsel et al., 2013). In contrast, the Shiga toxin produced by *Shigella dysenteriae*, the cholera toxin produced by *Vibrio cholerae* and the exotoxin produced by *P. aeruginosa* rely on functional retrograde trafficking to enter the eukaryotic cell cytosol (Johannes and Popoff, 2008), indicating that the intracellular and extracellular pathogens have different strategies for interactions with host cells. Another effector, LegG1, activates the host cell GTPase, Ran on the LCV membrane, causing polymerization of microtubules, allowing the LCV to move along the microtubules in host cells. A mutant of LegG1 exhibited compromised intracellular growth (Rothmeier et al., 2013; Hilbi et al., 2014). Interestingly, although the *L. pneumophila* infection generally inhibits chemotaxis of amoebae and macrophage, this inhibition is relieved by LegG1 expression. This may help host cells acquire nutrients from the environment, which then benefits *L. pneumophila* allowing for intracellular multiplication (Simon et al., 2014). Furthermore, many T4SS effectors encoded by the *L. pneumophila* genome are eukaryotic-like proteins which are believed to have been acquired during residence in protozoa, and these eukaryotic-like proteins are used as virulence factors to modulate protist host cell functions (Lurie-Weinberger et al., 2010; Gomez-Valero et al., 2011, 2014). Most of these proteins encode motifs that are involved in protein–protein interactions and mimic host proteins, thereby allowing control of host machinery and intracellular replication. These motifs include F-box, U-box, ankyrin and serine threonine protein kinase motifs.

In addition to the T4SS, the type II secretion system (T2SS) is also important for intracellular survival of *L. pneumophila* in protozoa and macrophage (Rossier et al., 2004) and the more than 25 exoproteins secreted by the T2SS are effective in a host specific manner (Cianciotto, 2009). For example, a T2SS secreted chitinase is responsible for persistence during lung infection in a mouse model of infection, but is not involved in survival in the amoeba, *Hartmannella vermiformis* or human macrophage-like cells (DebRoy et al., 2006). A mutation in a novel T2SS-dependant exoprotein, NttA, reduces intracellular survival in *A. castellanii*, but not other host cells, while the RNase, SrnA, acyltransferase, PlaC and metalloprotease, ProA promote multiplication of *L. pneumophila* in *H. vermiformis* and *Naegleria lovaniensis* only (Tyson et al., 2013).

*L. pneumophila* has a quorum sensing (QS) system consisting of an autoinducer synthase, LqsA, two sensor kinases, LqsS and LqsT, and a response regulator, LqsR, that controls virulence, motility, filament production, natural competence and the switch between the replicative and infectious forms (Kessler et al., 2013; Schell et al., 2014, 2016b; Personnic et al., 2017). A mutant of LqsR is defective for intracellular growth in *A. castellanii, D. discoideum* and macrophage (Tiaden et al., 2007). Interestingly, the autoinducer produced by LqsA, as well as its homologous cholera autoinducer-1 (CAI-1), inhibits the cell migration of *D. discoideum* and macrophage in a T4SS-independent manner, suggesting that these autoinducers play a role in interkingdom signaling (Simon et al., 2015).

*Mycobacterium* spp., including the causal agent of tuberculosis, *Mycobacterium tuberculosis*, uses similar strategies to infect and replicate in bacteria-containing vacuoles, such as inhibition of phagosome maturation (Awuh and Flo, 2017). It has been reported that *Mycobacterium marinum* can inhibit phagosome-lysosome fusion by inducing host WASH complex dependent actin polymerization in *D. discoideum* and macrophage, although the bacterial effector is still unknown (Kolonko et al., 2014). In *S. enterica* serovar Typhimurium, virulence factors [i.e., *waaL* responsible for ligation of O-antigen to LPS, *invA* and *ssaD* essential structural components of type 3 secretion systems (T3SS), *clpV* chaperone essential for protein secretion by type VI secretion systems (T6SS), and PhoP/PhoQ two-component system] were shown to be involved in intracellular survival in *D. discoideum* (Riquelme et al., 2016). Interestingly, *F. tularensis*, the causal agent of tularemia, exhibits different survival mechanisms in macrophage and amoebae (Ozanic et al., 2015). In macrophages, *F. tularensis* escapes from acidified phagosomes to the cytosol and then proliferates, while in *H. vermiformis*, it replicates in non-acidified phagosomes (Santic et al., 2011). The Francisella pathogenicity island (FPI) encoding the T6SS is critical for survival in both hosts. One of the FPI proteins, IglC, a homolog of the T6SS Hcp protein (de Bruin et al., 2011), is responsible for escape from phagosome to cytosol (Santic et al., 2005) and invasion of non-phagocytic epithelial cells (Law et al., 2014). Mutants of IglC and the regulator MglA are defective for intracellular survival in both macrophage and *A. castellanii* (Lauriano et al., 2004).

Taken together, the intracellular pathogens possess many dual role factors that mediate their interactions with both protozoa and human phagocytes. Most of the similarities are found between amoebae and macrophage due to the conservative phagocytic process. Intracellular pathogens use surface factors to facilitate invasion and then rely on various effectors delivered by secretion systems to inhibit phagosome maturation, where many effectors are under precise regulation. These dual role mechanisms more likely evolved during the long-term interactions with protozoa in the environment, rather than during infections of human and animals. The temporal and spatial distribution of opportunistic pathogens in the environment between hosts, provides greater chances for them to face grazing pressure by bacterivorous protozoa. As a result, humans and animals become the accidental victims of coincidental evolution and further support the coincidental evolution hypothesis.

## Extracellular Pathogens

**FIGURE 2:**
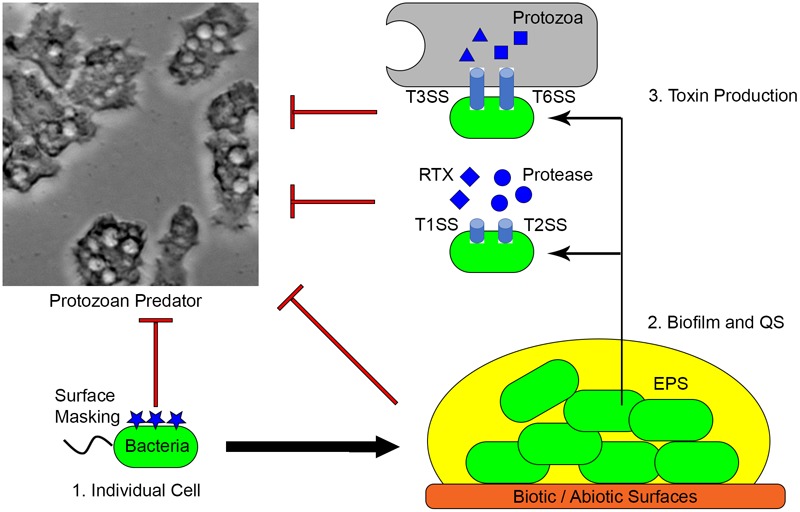
Mechanisms utilized by extracellular pathogens. (1) Surface masking facilitates bacterial cells avoiding phagocytosis. (2) Extracellular pathogens form biofilms on various surfaces. EPS production and QS-regulated activities protect bacteria from predation. (3) Toxins produced by the biofilm community are important for both grazing resistance and animal infection.

*V. cholerae* is the causal agent of cholera and is generally recognized as a model for non-invasive diarrheal disease (Sack et al., 2004). *V. cholerae* uses surface masking, toxin secretion and biofilm formation to defend against protozoan grazing and these mechanisms are also important for colonization and infection of humans (**Figure 2**) (Lutz et al., 2013). More than 200 serogroups classified by the lipopolysaccharide (LPS) O-antigen of *V. cholerae* exist, but only O1 and O139 have caused pandemics to date (Chatterjee and Chaudhuri, 2003). The *V. cholerae* LPS, or endotoxin, is an important virulence factor that mediates intestinal attachment and modulates host immunological responses (Chatterjee and Chaudhuri, 2006). The lipid-A of LPS has been proposed to inhibit phagocytosis by *T. pyriformis* (Kovacs et al., 1986). Similarly, production of LPS, capsular polysaccharide and outer membrane proteins protects *Klebsiella pneumoniae* from phagocytosis by *D. discoideum* and macrophage. Mutant strains of *K. pneumoniae* defective in the LPS core, lipid A, palmitoylation, OmpA and OmpK36 are susceptible to phagocytosis (March et al., 2013).

*V. cholerae* possesses multiple secretion systems that are important for pathogenesis and ecological fitness. The type I secretion system (T1SS) is responsible for delivery of RtxA, a multifunctional autoprocessing repeats-in-toxin (MARTX) toxin, which is involved in the colonization of the small intestine (Olivier et al., 2007) and destruction of host cell actin cytoskeleton (Prochazkova et al., 2009). MARTX is also secreted by the T1SS of *Vibrio vulnificus* and has cytopathogenic activity toward host cells, leading to rounding, cell death and interference of internalization by host phagocytes (Lee et al., 2007; Kim et al., 2008). *V. vulnificus* RtxA1 can cause damage in a variety of ways, including generation of reactive oxygen species (ROS) (Chung et al., 2010), cytoskeletal rearrangement (Kim et al., 2008), apoptotic cell death (Lee et al., 2008), and interference with the cytosolic Ca^2+^ flux to inhibit phagocytosis (Kuo et al., 2015). *V. vulnificus* produces at least four different types of MARTX (types I–IV), where the plasmid-encoded MARTX type III (RtxA13) is structurally and evolutionarily different to MARTX types I and II (Kwak et al., 2011; Roig et al., 2011). MARTX type III of *V. vulnificus* biotype 2 is involved in the lysis of a wide range of eukaryotic cells, including amoebae, erythrocytes, epithelial cells, and phagocytes. For example, the amoebae *Neoparamoeba pemaquidensis* isolated and purified from turbot (*Scophthalmus maximus*) gills, grew significantly less in the presence of the wild-type strain compared to the *rtxA1_3_* mutant (Lee et al., 2013).

The T2SS of *V. cholerae* is involved in the secretion of cholera toxin (Sandkvist et al., 1997). A recent study has reported that *V. cholerae* protease, PrtV, is transported across the outer membrane by T2SS and then packed into outer membrane vesicles (Rompikuntal et al., 2015). *V. cholerae* PrtV, which causes instant cytotoxic effects during infection in human intestinal cell lines, is also toxic to predators such as the ciliate *T. pyriformis* and the flagellate *Cafeteria roenbergensis* (Vaitkevicius et al., 2006).

Although there are limited studies of T3SS-dependent virulence factors in *V. cholerae*, including VopE (Suzuki et al., 2014), VopF (Tam et al., 2007), and VopX (Alam et al., 2011), and the role of *V. cholerae* T3SS in grazing resistance has not been reported, T3SS has been shown to contribute to anti-protozoal activities in *E. coli* (Siddiqui et al., 2011), *P. aeruginosa* (Matz et al., 2008) and *Vibrio parahaemolyticus* (Matz et al., 2011). Interestingly, pathogenic *E. coli* strains (EHEC or STEC) encode a second T3SS, which may not be a functional secretion system, but is still important for virulence during infection and may be associated with Shiga toxin (Zhou et al., 2014). Shiga toxin is the major virulence factor in EHEC strains, but its role in grazing resistance is under debate. It has been reported that Shiga toxins of *E. coli* O157:H7 provides a survival advantage in *T. pyriformis* food vacuoles (Steinberg and Levin, 2007) and is cytotoxic to *A. castellanii* (Arnold and Koudelka, 2014) and *T. thermophila* (Stolfa and Koudelka, 2012). In contrast, there are also studies showing that Shiga toxin is not important in association of EHEC strains with *A. castellanii* (Chekabab et al., 2012) and may even decrease survival in the presence of *A. castellanii* (Chekabab et al., 2013). Virulence of EHEC strains depends on the induction of Shiga toxin-converting prophage. Bacteriophage-mediated lysis of Shiga toxin-encoding bacteria is necessary for toxicity in protozoa whereas digestion of bacteria by *T. thermophila* is harmless to the cell. Shiga toxin-converting prophage can be induced via treatment with H_2_O_2_. EHEC strains with mutations in bacterial SOS response genes exhibit increased susceptibility to grazing by *T. thermophila* (Lainhart et al., 2009; Stolfa and Koudelka, 2012). Oxidative stress as a consequence of H_2_O_2_ excretion by either neutrophils during infection or protist predators could potentially induce the production of Shiga toxin-converting prophage (Licznerska et al., 2016). A phage encoded toxin is also important for the anti-protozoal activity of *Corynebacterium diphtheriae*, as the diphtheria toxin kills *A. castellanii* during co-culturing (Arnold and Koudelka, 2014).

The T6SS of *V. cholerae* injects effector proteins into target cells by the action of a structure analogous to an intracellular membrane-attached contractile phage tail sheath (Basler et al., 2012; Ho et al., 2014). In *V. cholerae* non-O1/non-O139 strains, the T6SS has been shown to have toxic effects toward *D. discoideum* as well as mammalian macrophages (Pukatzki et al., 2006, 2007; Miyata et al., 2011). In addition to the direct role of T6SS in eukaryotic host pathogenesis by the injection of toxins responsible for actin crosslinking, the T6SS also has an indirect role in competition with neighboring bacteria (Dong et al., 2013; Ho et al., 2014).

Biofilm formation is important for both pathogenesis and environmental survival. Colonization of intestinal epithelial cells allows *V. cholerae* to deliver virulence factors effectively and provides resistance against various stresses, such as bile acids and antimicrobial peptides (Silva and Benitez, 2016). Biofilm formation also protects *V. cholerae* against predation while planktonic cells are eliminated. In addition, predation by protozoa promotes *V. cholerae* biofilm formation and induces a smooth to rugose morphotypic shift due to increased extracellular polysaccharide (EPS) production (Matz et al., 2005). EPS as the major component of the biofilm matrix, shields *V. cholerae* from predation by the surface feeding nanoflagellate, *Rhynchomonas nasuta* and *A. castellanii* (Sun et al., 2013). High cell densities within biofilms also allow accumulation of bioactive compounds. For example, production of pyomelanin plays a role in virulence factor expression and colonization by *V. cholerae* (Valeru et al., 2009). Pyomelanin production in *V. cholerae* biofilm also leads to increased ROS levels, which promotes resistance to predation by *A. castellanii* (Noorian et al., 2017).

The high cell density of *V. cholerae* biofilms enables QS, which regulates a number of virulence factors including motility, protease secretion, toxin production via the master regulator ToxR and biofilm formation (Zhu et al., 2002). Both PrtV and the T6SS are QS-regulated. A mutant in the *V. cholerae* QS master response regulator, *hapR*, resulted in reduced grazing resistance in comparison to the wild-type when exposed to *R. nasuta* (Matz et al., 2005). When biofilms of mixed *V. cholerae* wild-type and QS-deficient strains were exposed to predation, the QS mutant was selectively removed, indicating that QS also regulates *V. cholerae* surface components recognized by protozoa (Sun et al., 2013).

Similar to *V. cholerae*, biofilm formation increases *P. aeruginosa* resistance to host defenses and chemotherapy (Mulcahy et al., 2014). In addition, EPS and QS-regulated inhibitors are also responsible for grazing resistance of *P. aeruginosa* biofilms (Matz et al., 2004; Weitere et al., 2005). A newly identified *P. aeruginosa* modulator, GTPase TypA, is responsible for surface attachment and biofilm formation, resistance to antibiotics, and T3SS-dependent virulence. A mutant of TypA showed reduced resistance to *D. discoideum* grazing and increased uptake by macrophage (Neidig et al., 2013).

In contrast to intracellular pathogens, extracellular pathogens use surface factors to avoid internalization and produce various toxins to inhibit protozoan predators. Biofilm formation provides physical protection and accumulates anti-protozoal compounds. These dual role factors also enable extracellular pathogens to colonize epithelial surfaces and deliver toxins to host cells in an effective manner. Notably, the host cell receptors and targets of many dual role toxins are not always conserved between protozoa and human phagocytes, but nonetheless, are effective in both instances. Thus, toxin production can lead to enhanced virulence during infections.

## Coincidental Evolution Hypothesis

The coincidental evolution hypothesis suggests that virulence factors arose as a response to other selective pressures, such as predation, rather than for virulence *per se* (Levin, 1996; Adiba et al., 2010; Brown et al., 2012; Erken et al., 2013). There are many examples of virulence factors or their homologs that exist in environmental microorganisms that potentially play another functional role in the environment, including but not limited to grazing resistance. In the case of *V. cholerae*, it has been demonstrated that the T6SS of *V. cholerae* is functionally activated under high osmolarity and low temperature conditions, suggesting that the system may be important for the survival of the bacterium in the environment (Ishikawa et al., 2012). Furthermore, the cold shock gene, *cspV*, which controls biofilm formation through modulation of the second messenger cyclic-di-GMP, also regulates T6SS-mediated interspecies killing in a temperature-dependent manner (Townsley et al., 2016). A mutant in *cspV* showed significant defects for attachment and T6SS-mediated killing on the surface of the aquatic crustacean *Daphnia magna* (Townsley et al., 2016). In addition, chitin colonization is important for the long-term environmental persistence of *V. cholerae* (Pruzzo et al., 2008), where *V. cholerae* colonization factor GbpA (Kirn et al., 2005; Wong et al., 2012) and toxin co-regulated pilus (Reguera and Kolter, 2005) are involved in attachment to both chitin and epithelial surfaces.

The evolution of virulence driven by protozoan grazing has also been reported for fungi and viruses. *Cryptococcus neoformans* is a yeast that can cause lung infections in immunocompromised people and invades the brain by using host monocytes as “Trojan horses” (Charlier et al., 2009). Its ability to survive intracellularly has been proposed to have evolved for protection against amoeba predation (Steenbergen et al., 2001; Casadevall, 2012). Although the mechanisms of association are still unknown, *A. castellanii* has been reported to be the carrier or vectors for at least 2 human viruses, adenovirus (Scheid and Schwarzenberger, 2012) and coxsackie B3 virus (Mattana et al., 2006). For coxsackie B3 virus, an enrichment on *A. castellanii* surfaces was observed and the virus was eventually located inside the trophozoite (Mattana et al., 2006).

In some cases, grazing resistance mechanisms benefit the bacterium in association with a broad range of hosts. The methionine sulfoxide reductase can repair ROS-damaged proteins and are important for many bacterial pathogens (Sasindran et al., 2007). Mutation of methionine sulfoxide reductases in *Aeromonas hydrophila* reduces resistance to predation by *T. thermophila* and infection of zebrafish (Pang et al., 2016). Hemagglutinin protease which destroys host cell receptors for several different *V. cholerae* adhesins, is also involved in the degradation of chironomid egg masses (Halpern et al., 2003). Furthermore, QS-regulated production of hemagglutinin protease is important for defense against phages within high cell density bacterial populations (Hoque et al., 2016).

The bacterial–protozoal interaction has been suggested to be one of the oldest interactions between prokaryotic and eukaryotic organisms (Cavalier-Smith, 2002). During their long history of interaction, many virulence factors of bacterial pathogens evolved as adaptations to grazing pressure, rather than for pathogenesis to human and animal hosts. For example, a genome analysis of a *Chlamydia*-related amoebal symbiont indicates that many traits for intracellular survival, including the T3SS, existed in the bacterial genome 700 million years ago, 500 million years earlier than the appearance of Mammalia (Horn et al., 2004).

Our understanding of the pathogenesis of intracellular and extracellular pathogens is distorted due to our tendency to focus on the pathogen in the human host rather than in their natural environmental niche. The functions of virulence genes or their homologs in the environment have for the most part, not been fully explored. Here, we summarized the bacterial mechanisms providing grazing resistance and virulence in model intracellular and extracellular pathogens. For intracellular pathogens, active invasion, blocking of the phagosome lysosome fusion and resistance to phagocytic digestion are critical traits for infecting host cells. Extracellular pathogens, in contrast, rely on toxin secretion and biofilm formation as important mechanisms of protection against predation and pathogenesis. Some bacteria possess traits common in both intracellular and extracellular pathogens. For example, *L. pneumophila* can develop biofilms on environmental surfaces (Abdel-Nour et al., 2013) and *V. cholerae* has been found to proliferate inside of amoebae (Abd et al., 2005; Van der Henst et al., 2015). Since bacterial surface components are involved in the initial contact with host cells, possession of factors mediating active invasion may be used as a key feature to distinguish intracellular and extracellular pathogens. In conclusion, evolution of mechanisms that allow for survival within protozoa may have selected for traits that also allow bacteria to escape that harmful effects of phagocytes.

## Author Contributions

SS, PN, and DM contributed to the writing of the first draft of the manuscript. SS, PN, and DM contributed to manuscript revision, read and approved the submitted version.

## Conflict of Interest Statement

The authors declare that the research was conducted in the absence of any commercial or financial relationships that could be construed as a potential conflict of interest.

## References

[B1] Abd H. Weintraub A. Sandstrom G. (2005). Intracellular survival and replication of *Vibrio cholerae* O139 in aquatic free-living amoebae. 7 1003–1008. 10.1111/j.1462-2920.2005.00771.x 15946296

[B2] Abdel-Nour M. Duncan C. Low D. E. Guyard C. (2013). Biofilms: the stronghold of *Legionella pneumophila*. 14 21660–21675. 10.3390/ijms141121660 24185913 PMC3856027

[B3] Adiba S. Nizak C. van Baalen M. Denamur E. Depaulis F. (2010). From grazing resistance to pathogenesis: the coincidental evolution of virulence factors. 5:e11882. 10.1371/journal.pone.0011882 20711443 PMC2920306

[B4] Aijaz I. Koudelka G. B. (2017). *Tetrahymena* phagocytic vesicles as ecological micro-niches of phage transfer. 93:fix030. 10.1093/femsec/fix030 28334205

[B5] Alam A. Miller K. A. Chaand M. Butler J. S. Dziejman M. (2011). Identification of *Vibrio cholerae* type III secretion system effector proteins. 79 1728–1740. 10.1128/IAI.01194-10 21282418 PMC3067535

[B6] Arnold J. W. Koudelka G. B. (2014). The Trojan Horse of the microbiological arms race: phage-encoded toxins as a defence against eukaryotic predators. 16 454–466. 10.1111/1462-2920.12232 23981100

[B7] Awuh J. A. Flo T. H. (2017). Molecular basis of mycobacterial survival in macrophages. 74 1625–1648. 10.1007/s00018-016-2422-8 27866220 PMC11107535

[B8] Azumah B. K. Addo P. G. Dodoo A. Awandare G. Mosi L. Boakye D. A. (2017). Experimental demonstration of the possible role of *Acanthamoeba polyphaga* in the infection and disease progression in Buruli Ulcer (BU) using ICR mice. 12:e0172843. 10.1371/journal.pone.0172843 28329001 PMC5362167

[B9] Balczun C. Scheid P. L. (2017). Free-living amoebae as hosts for and vectors of intracellular microorganisms with public health significance. 9:E65. 10.3390/v9040065 28368313 PMC5408671

[B10] Barker J. Brown M. R. (1994). Trojan horses of the microbial world: protozoa and the survival of bacterial pathogens in the environment. 140(Pt 6) 1253–1259. 10.1099/00221287-140-6-1253 8081490

[B11] Basler M. Pilhofer M. Henderson G. P. Jensen G. J. Mekalanos J. J. (2012). Type VI secretion requires a dynamic contractile phage tail-like structure. 483 182–186. 10.1038/nature10846 22367545 PMC3527127

[B12] Boamah D. K. Zhou G. Ensminger A. W. O’Connor T. J. (2017). From many hosts, one accidental pathogen: the diverse protozoan hosts of *Legionella*. 7:477. 10.3389/fcimb.2017.00477 29250488 PMC5714891

[B13] Brown S. P. Cornforth D. M. Mideo N. (2012). Evolution of virulence in opportunistic pathogens: generalism, plasticity, and control. 20 336–342. 10.1016/j.tim.2012.04.005 22564248 PMC3491314

[B14] Burgess S. L. Gilchrist C. A. Lynn T. C. Petri W. A. (2017). Parasitic protozoa and interactions with the host intestinal microbiota. 85:e00101-17. 10.1128/IAI.00101-17 28584161 PMC5520446

[B15] Buse H. Y. Schaefer F. W. III Rice E. W. (2017). Enhanced survival but not amplification of *Francisella* spp. in the presence of free-living amoebae. 64 17–36. 10.1556/030.63.2016.015 27929353 PMC7357732

[B16] Butt A. T. Thomas M. S. (2017). Iron acquisition mechanisms and their role in the virulence of *Burkholderia* species. 7:460. 10.3389/fcimb.2017.00460 29164069 PMC5681537

[B17] Carlson S. A. Sharma V. K. McCuddin Z. P. Rasmussen M. A. Franklin S. K. (2007). Involvement of a *Salmonella* genomic island 1 gene in the rumen protozoan-mediated enhancement of invasion for multiple-antibiotic-resistant *Salmonella enterica* serovar Typhimurium. 75 792–800. 10.1128/IAI.00679-06 17145942 PMC1828496

[B18] Casadevall A. (2012). Amoeba provide insight into the origin of virulence in pathogenic fungi. 710 1–10. 10.1007/978-1-4419-5638-5_1 22127880

[B19] Cateau E. Maisonneuve E. Peguilhan S. Quellard N. Hechard Y. Rodier M. H. (2014). *Stenotrophomonas* maltophilia and *Vermamoeba vermiformis* relationships: bacterial multiplication and protection in amoebal-derived structures. 165 847–851. 10.1016/j.resmic.2014.10.004 25463386

[B20] Cavalier-Smith T. (2002). The phagotrophic origin of eukaryotes and phylogenetic classification of Protozoa. 52 297–354. 10.1099/00207713-52-2-297 11931142

[B21] Cervero-Arago S. Rodriguez-Martinez S. Puertas-Bennasar A. Araujo R. M. (2015). Effect of common drinking water disinfectants, chlorine and heat, on free *Legionella* and amoebae-associated *Legionella*. 10:e0134726. 10.1371/journal.pone.0134726 26241039 PMC4524690

[B22] Cervero-Arago S. Sommer R. Araujo R. M. (2014). Effect of UV irradiation (253.7 nm) on free *Legionella* and *Legionella* associated with its amoebae hosts. 67 299–309. 10.1016/j.watres.2014.09.023 25306486

[B23] Chabe M. Lokmer A. Segurel L. (2017). Gut protozoa: friends or foes of the human gut microbiota? 33 925–934. 10.1016/j.pt.2017.08.005 28870496

[B24] Charlier C. Nielsen K. Daou S. Brigitte M. Chretien F. Dromer F. (2009). Evidence of a role for monocytes in dissemination and brain invasion by *Cryptococcus neoformans*. 77 120–127. 10.1128/IAI.01065-08 18936186 PMC2612285

[B25] Chatterjee S. N. Chaudhuri K. (2003). Lipopolysaccharides of *Vibrio cholerae*. I. Physical and chemical characterization. 1639 65–79. 10.1016/j.bbadis.2003.08.004 14559113

[B26] Chatterjee S. N. Chaudhuri K. (2006). Lipopolysaccharides of *Vibrio* cholerae: III. Biological functions. 1762 1–16. 10.1016/j.bbadis.2005.08.005 16185850

[B27] Chekabab S. M. Daigle F. Charette S. J. Dozois C. M. Harel J. (2012). Survival of enterohemorrhagic *Escherichia coli* in the presence of *Acanthamoeba castellanii* and its dependence on Pho regulon. 1 427–437. 10.1002/mbo3.40 23233434 PMC3535388

[B28] Chekabab S. M. Daigle F. Charette S. J. Dozois C. M. Harel J. (2013). Shiga toxins decrease enterohaemorrhagic *Escherichia coli* survival within *Acanthamoeba castellanii*. 344 86–93. 10.1111/1574-6968.12158 23581502

[B29] Chung K.-J. Cho E.-J. Kim M. K. Kim Y. R. Kim S.-H. Yang H.-Y. (2010). RtxA1-induced expression of the small GTPase Rac2 plays a key role in the pathogenicity of *Vibrio vulnificus*. 201 97–105. 10.1086/648612 19919301

[B30] Cianciotto N. P. (2009). Many substrates and functions of type II secretion: lessons learned from *Legionella pneumophila*. 4 797–805. 10.2217/fmb.09.53 19722835 PMC2754693

[B31] Cirillo J. D. Cirillo S. L. Yan L. Bermudez L. E. Falkow S. Tompkins L. S. (1999). Intracellular growth in *Acanthamoeba castellanii* affects monocyte entry mechanisms and enhances virulence of *Legionella pneumophila*. 67 4427–4434. 10456883 10.1128/iai.67.9.4427-4434.1999 PMC96761

[B32] Danelishvili L. Wu M. Stang B. Harriff M. Cirillo S. L. Cirillo J. D. (2007). Identification of *Mycobacterium avium* pathogenicity island important for macrophage and amoeba infection. 104 11038–11043. 10.1073/pnas.0610746104 17578930 PMC1904132

[B33] de Bruin O. M. Duplantis B. N. Ludu J. S. Hare R. F. Nix E. B. Schmerk C. L. (2011). The biochemical properties of the *Francisella* pathogenicity island (FPI)-encoded proteins IglA, IglB, IglC, PdpB and DotU suggest roles in type VI secretion. 157 3483–3491. 10.1099/mic.0.052308-0 21980115 PMC3352279

[B34] DebRoy S. Dao J. Soderberg M. Rossier O. Cianciotto N. P. (2006). *Legionella pneumophila* type II secretome reveals unique exoproteins and a chitinase that promotes bacterial persistence in the lung. 103 19146–19151. 10.1073/pnas.0608279103 17148602 PMC1748190

[B35] Denoncourt A. M. Paquet V. E. Charette S. J. (2014). Potential role of bacteria packaging by protozoa in the persistence and transmission of pathogenic bacteria. 5:240. 10.3389/fmicb.2014.00240 24904553 PMC4033053

[B36] Dietrich C. Heuner K. Brand B. C. Hacker J. Steinert M. (2001). Flagellum of *Legionella pneumophila* positively affects the early phase of infection of eukaryotic host cells. 69 2116–2122. 10.1128/IAI.69.4.2116-2122.2001 11254565 PMC98137

[B37] Dong T. G. Ho B. T. Yoder-Himes D. R. Mekalanos J. J. (2013). Identification of T6SS-dependent effector and immunity proteins by Tn-seq in *Vibrio cholerae*. 110 2623–2628. 10.1073/pnas.1222783110 23362380 PMC3574944

[B38] Erken M. Lutz C. McDougald D. (2013). The rise of pathogens: Predation as a factor driving the evolution of human pathogens in the environment. 65 860–868. 10.1007/s00248-013-0189-0 23354181 PMC3637895

[B39] Escoll P. Rolando M. Gomez-Valero L. Buchrieser C. (2013). From amoeba to macrophages: exploring the molecular mechanisms of *Legionella pneumophila* infection in both hosts. 376 1–34. 10.1007/82_2013_351 23949285

[B40] Fenchel T. (1987). *Ecology of Protozoa: The Biology of Free-Living Phagotropic Protists*. Berlin: Springer.

[B41] Fieseler L. Doyscher D. Loessner M. J. Schuppler M. (2014). *Acanthamoeba* release compounds which promote growth of *Listeria monocytogenes* and other bacteria. 98 3091–3097. 10.1007/s00253-014-5534-9 24562324

[B42] Finsel I. Hilbi H. (2015). Formation of a pathogen vacuole according to *Legionella pneumophila*: how to kill one bird with many stones. 17 935–950. 10.1111/cmi.12450 25903720

[B43] Finsel I. Ragaz C. Hoffmann C. Harrison C. F. Weber S. van Rahden V. A. (2013). The *Legionella* effector RidL inhibits retrograde trafficking to promote intracellular replication. 14 38–50. 10.1016/j.chom.2013.06.001 23870312

[B44] German N. Doyscher D. Rensing C. (2013). Bacterial killing in macrophages and amoeba: do they all use a brass dagger? 8 1257–1264. 10.2217/fmb.13.100 24059917

[B45] Gomez-Valero L. Rusniok C. Cazalet C. Buchrieser C. (2011). Comparative and functional genomics of Legionella identified eukaryotic like proteins as key players in host-pathogen interactions. 2:208. 10.3389/fmicb.2011.00208 22059087 PMC3203374

[B46] Gomez-Valero L. Rusniok C. Rolando M. Neou M. Dervins-Ravault D. Demirtas J. (2014). Comparative analyses of *Legionella* species identifies genetic features of strains causing Legionnaires’ disease. 15:505. 25370836 10.1186/s13059-014-0505-0 PMC4256840

[B47] Gong J. Qing Y. Zou S. Fu R. Su L. Zhang X. (2016). Protist-bacteria associations: *Gammaproteobacteria* and *Alphaproteobacteria* are prevalent as digestion-resistant bacteria in ciliated protozoa. 7:498. 10.3389/fmicb.2016.00498 27148188 PMC4826875

[B48] Halpern M. Gancz H. Broza M. Kashi Y. (2003). *Vibrio cholerae* hemagglutinin/protease degrades chironomid egg masses. 69 4200–4204. 10.1128/AEM.69.7.4200-4204.2003 12839800 PMC165141

[B49] Hao X. Luthje F. Ronn R. German N. A. Li X. Huang F. (2016). A role for copper in protozoan grazing - two billion years selecting for bacterial copper resistance. 102 628–641. 10.1111/mmi.13483 27528008

[B50] Hilbi H. Rothmeier E. Hoffmann C. Harrison C. F. (2014). Beyond Rab GTPases Legionella activates the small GTPase Ran to promote microtubule polymerization, pathogen vacuole motility, and infection. 5 1–6. 10.4161/21541248.2014.972859 25496424 PMC4601303

[B51] Ho B. T. Dong T. G. Mekalanos J. J. (2014). A view to a kill: the bacterial type VI secretion system. 15 9–21. 10.1016/j.chom.2013.11.008 24332978 PMC3936019

[B52] Hoffmann C. Harrison C. F. Hilbi H. (2014). The natural alternative: protozoa as cellular models for *Legionella* infection. 16 15–26. 10.1111/cmi.12235 24168696

[B53] Hood M. I. Skaar E. P. (2012). Nutritional immunity: transition metals at the pathogen–host interface. 10 525–537. 10.1038/nrmicro2836 22796883 PMC3875331

[B54] Hoppe J. Unal C. M. Thiem S. Grimpe L. Goldmann T. Gassler N. (2017). Pily1 promotes *Legionella pneumophila* infection of human lung tissue explants and contributes to bacterial adhesion, host cell invasion, and twitching motility. 7:63. 10.3389/fcimb.2017.00063 28326293 PMC5339237

[B55] Hoque M. M. Naser I. B. Bari S. M. N. Zhu J. Mekalanos J. J. Faruque S. M. (2016). Quorum regulated resistance of *Vibrio cholerae* against environmental bacteriophages. 6:37956. 10.1038/srep37956 27892495 PMC5124996

[B56] Horn M. Collingro A. Schmitz-Esser S. Beier C. L. Purkhold U. Fartmann B. (2004). Illuminating the evolutionary history of chlamydiae. 304 728–730. 10.1126/science.1096330 15073324

[B57] Ishikawa T. Sabharwal D. Bröms J. Milton D. L. Sjöstedt A. Uhlin B. E. (2012). Pathoadaptive conditional regulation of the Type VI secretion system in *Vibrio cholerae* O1 strains. 80 575–584. 10.1128/IAI.05510-11 22083711 PMC3264300

[B58] Johannes L. Popoff V. (2008). Tracing the retrograde route in protein trafficking. 135 1175–1187. 10.1016/j.cell.2008.12.009 19109890

[B59] Juhas M. (2015). Horizontal gene transfer in human pathogens. 41 101–108. 10.3109/1040841X.2013.804031 23862575

[B60] Jurgens K. Matz C. (2002). Predation as a shaping force for the phenotypic and genotypic composition of planktonic bacteria. 81 413–434. 10.1023/A:1020505204959 12448740

[B61] Kessler A. Schell U. Sahr T. Tiaden A. Harrison C. Buchrieser C. (2013). The *Legionella pneumophila* orphan sensor kinase LqsT regulates competence and pathogen-host interactions as a component of the LAI-1 circuit. 15 646–662. 10.1111/j.1462-2920.2012.02889.x 23033905

[B62] Kilvington S. Price J. (1990). Survival of *Legionella pneumophila* within cysts of *Acanthamoeba polyphaga* following chlorine exposure. 68 519–525. 10.1111/j.1365-2672.1990.tb02904.x 2196257

[B63] Kim Y. R. Lee S. E. Kook H. Yeom J. A. Na H. S. Kim S. Y. (2008). *Vibrio vulnificus* RTX toxin kills host cells only after contact of the bacteria with host cells. 10 848–862. 10.1111/j.1462-5822.2007.01088.x 18005241

[B64] Kirn T. J. Jude B. A. Taylor R. K. (2005). A colonization factor links *Vibrio cholerae* environmental survival and human infection. 438 863–866. 10.1038/nature04249 16341015

[B65] Kolonko M. Geffken A. C. Blumer T. Hagens K. Schaible U. E. Hagedorn M. (2014). WASH-driven actin polymerization is required for efficient mycobacterial phagosome maturation arrest. 16 232–246. 10.1111/cmi.12217 24119059

[B66] Kovacs G. Nagy S. U. Csaba G. (1986). The effect of bacterial endotoxin of phagocytosis of Tetrahymena and serotonin induced imprinting. 33 301–304. 3307273

[B67] Kuo S. Y. Chou M. C. Lee S. L. Wang Y. Chen C. L. Lin P. T. (2015). Vibrio vulnificus RtxA1 modulated calcium flux contributes reduced internalization in phagocytes. 132 55–60. 10.1016/j.lfs.2015.03.027 25916802

[B68] Kwak J. S. Jeong H.-G. Satchell K. J. F. (2011). Vibrio vulnificus rtxA1 gene recombination generates toxin variants with altered potency during intestinal infection. 108 1645–1650. 10.1073/pnas.1014339108 21220343 PMC3029691

[B69] Lainhart W. Stolfa G. Koudelka G. B. (2009). Shiga toxin as a bacterial defense against a eukaryotic predator, *Tetrahymena thermophila*. 191 5116–5122. 10.1128/JB.00508-09 19502393 PMC2725575

[B70] Lambrecht E. Bare J. Chavatte N. Bert W. Sabbe K. Houf K. (2015). Protozoan cysts act as a survival niche and protective shelter for foodborne pathogenic bacteria. 81 5604–5612. 10.1128/AEM.01031-15 26070667 PMC4510183

[B71] Lambrecht E. Bare J. Van Damme I. Bert W. Sabbe K. Houf K. (2013). Behavior of *Yersinia enterocolitica* in the presence of the bacterivorous *Acanthamoeba castellanii*. 79 6407–6413. 10.1128/AEM.01915-13 23934496 PMC3811209

[B72] Lauriano C. M. Barker J. R. Yoon S. S. Nano F. E. Arulanandam B. P. Hassett D. J. (2004). MglA regulates transcription of virulence factors necessary for *Francisella tularensis* intraamoebae and intramacrophage survival. 101 4246–4249. 10.1073/pnas.0307690101 15010524 PMC384726

[B73] Law H. T. Sriram A. Fevang C. Nix E. B. Nano F. E. Guttman J. A. (2014). IglC and PdpA are important for promoting *Francisella* invasion and intracellular growth in epithelial cells. 9:e104881. 10.1371/journal.pone.0104881 25115488 PMC4130613

[B74] Lee B. C. Choi S. H. Kim T. S. (2008). *Vibrio vulnificus* RTX toxin plays an important role in the apoptotic death of human intestinal epithelial cells exposed to *Vibrio vulnificus*. 10 1504–1513. 10.1016/j.micinf.2008.09.006 18849006

[B75] Lee C. T. Pajuelo D. Llorens A. Chen Y. H. Leiro J. M. Padrós F. (2013). MARTX of Vibrio vulnificus biotype 2 is a virulence and survival factor. 15 419–432. 10.1111/j.1462-2920.2012.02854.x 22943291

[B76] Lee J. H. Kim M. W. Kim B. S. Kim S. M. Lee B. C. Kim T. S. (2007). Identification and characterization of the *Vibrio vulnificus* rtxA essential for cytotoxicity in vitro and virulence in mice. 45 146–152. 17483800

[B77] Levin B. R. (1996). The evolution and maintenance of virulence in microparasites. 2 93–102. 10.3201/eid0202.960203 8903208 PMC2639826

[B78] Licznerska K. Nejman-Faleńczyk B. Bloch S. Dydecka A. Topka G. Gąsior T. (2016). Oxidative stress in shiga toxin production by enterohemorrhagic *Escherichia coli*. 2016:8. 10.1155/2016/3578368 26798420 PMC4699097

[B79] Lurie-Weinberger M. N. Gomez-Valero L. Merault N. Glockner G. Buchrieser C. Gophna U. (2010). The origins of eukaryotic-like proteins in *Legionella pneumophila*. 300 470–481. 10.1016/j.ijmm.2010.04.016 20537944

[B80] Lutz C. Erken M. Noorian P. Sun S. McDougald D. (2013). Environmental reservoirs and mechanisms of persistence of *Vibrio cholerae*. 4:375 10.3389/fmicb.2013.00375 PMC3863721 24379807

[B81] Madsen J. S. Burmolle M. Hansen L. H. Sorensen S. J. (2012). The interconnection between biofilm formation and horizontal gene transfer. 65 183–195. 10.1111/j.1574-695X.2012.00960.x 22444301

[B82] March C. Cano V. Moranta D. Llobet E. Perez-Gutierrez C. Tomas J. M. (2013). Role of bacterial surface structures on the interaction of *Klebsiella pneumoniae* with phagocytes. 8:e56847. 10.1371/journal.pone.0056847 23457627 PMC3574025

[B83] Mattana A. Serra C. Mariotti E. Delogu G. Fiori P. L. Cappuccinelli P. (2006). *Acanthamoeba castellanii* promotion of in vitro survival and transmission of coxsackie B3 viruses. 5 665–671. 10.1128/EC.5.4.665-671.2006 16607014 PMC1459673

[B84] Matz C. Bergfeld T. Rice S. A. Kjelleberg S. (2004). Microcolonies, quorum sensing and cytotoxicity determine the survival of *Pseudomonas aeruginosa* biofilms exposed to protozoan grazing. 6 218–226. 10.1111/j.1462-2920.2004.00556.x 14871206

[B85] Matz C. Kjelleberg S. (2005). Off the hook–how bacteria survive protozoan grazing. 13 302–307. 10.1016/j.tim.2005.05.009 15935676

[B86] Matz C. McDougald D. Moreno A. M. Yung P. Y. Yildiz F. H. Kjelleberg S. (2005). Biofilm formation and phenotypic variation enhance predation-driven persistence of *Vibrio cholerae*. 102 16819–16824. 10.1073/pnas.0505350102 16267135 PMC1283802

[B87] Matz C. Moreno A. M. Alhede M. Manefield M. Hauser A. R. Givskov M. (2008). *Pseudomonas aeruginosa* uses type III secretion system to kill biofilm-associated amoebae. 2 843–852. 10.1038/ismej.2008.47 18480848 PMC2662702

[B88] Matz C. Nouri B. McCarter L. Martinez-Urtaza J. (2011). Acquired type III secretion system determines environmental fitness of epidemic *Vibrio parahaemolyticus* in the interaction with bacterivorous protists. 6:e20275. 10.1371/journal.pone.0020275 21629787 PMC3100340

[B89] Miyata S. T. Kitaoka M. Brooks T. M. McAuley S. B. Pukatzki S. (2011). *Vibrio cholerae* requires the Type VI Secretion system virulence factor VasX to kill *Dictyostelium discoideum*. 79 2941–2949. 10.1128/IAI.01266-10 21555399 PMC3191968

[B90] Molmeret M. Horn M. Wagner M. Santic M. Abu Kwaik Y. (2005). Amoebae as training grounds for intracellular bacterial pathogens. 71 20–28. 10.1128/AEM.71.1.20-28.2005 15640165 PMC544274

[B91] Mulcahy L. R. Isabella V. M. Lewis K. (2014). *Pseudomonas aeruginosa* biofilms in disease. 68 1–12. 10.1007/s00248-013-0297-x 24096885 PMC3977026

[B92] Neidig A. Yeung A. T. Rosay T. Tettmann B. Strempel N. Rueger M. (2013). TypA is involved in virulence, antimicrobial resistance and biofilm formation in *Pseudomonas aeruginosa*. 13:77. 10.1186/1471-2180-13-77 23570569 PMC3639842

[B93] Noorian P. Hu J. Chen Z. Kjelleberg S. Wilkins M. R. Sun S. (2017). Pyomelanin produced by *Vibrio cholerae* confers resistance to predation by *Acanthamoeba castellanii*. 93:fix147. 10.1093/femsec/fix147 29095994 PMC5812506

[B94] Olivier V. Salzman N. H. Satchell K. J. (2007). Prolonged colonization of mice by *Vibrio cholerae* El Tor O1 depends on accessory toxins. 75 5043–5051. 10.1128/IAI.00508-07 17698571 PMC2044531

[B95] Ozanic M. Marecic V. Abu Kwaik Y. Santic M. (2015). The divergent intracellular lifestyle of *Francisella tularensis* in evolutionarily distinct host cells. 11:e1005208. 10.1371/journal.ppat.1005208 26633893 PMC4669081

[B96] Pang M. Lin X. Liu J. Guo C. Gao S. Du H. (2016). Identification of *Aeromonas hydrophila* genes preferentially expressed after phagocytosis by *Tetrahymena* and involvement of methionine sulfoxide reductases. 6:199. 10.3389/fcimb.2016.00199 28083518 PMC5183988

[B97] Paquet V. E. Charette S. J. (2016). Amoeba-resisting bacteria found in multilamellar bodies secreted by *Dictyostelium discoideum*: social amoebae can also package bacteria. 92:fiw025. 10.1093/femsec/fiw025 26862140

[B98] Parry J. D. (2004). Protozoan grazing of freshwater biofilms. 54 167–196. 10.1016/S0065-2164(04)54007-8 15251281

[B99] Personnic N. Striednig B. Hilbi H. (2017). *Legionella* quorum sensing and its role in pathogen-host interactions. 41 29–35. 10.1016/j.mib.2017.11.010 29190490

[B100] Portier E. Zheng H. Sahr T. Burnside D. M. Mallama C. Buchrieser C. (2015). IroT/mavN, a new iron-regulated gene involved in *Legionella pneumophila* virulence against amoebae and macrophages. 17 1338–1350. 10.1111/1462-2920.12604 25141909 PMC4672373

[B101] Prochazkova K. Shuvalova L. A. Minasov G. Voburka Z. Anderson W. F. Satchell K. J. (2009). Structural and molecular mechanism for autoprocessing of MARTX toxin of *Vibrio cholerae* at multiple sites. 284 26557–26568. 10.1074/jbc.M109.025510 19620709 PMC2785344

[B102] Pruzzo C. Vezzulli L. Colwell R. R. (2008). Global impact of *Vibrio cholerae* interactions with chitin. 10 1400–1410. 10.1111/j.1462-2920.2007.01559.x 18312392

[B103] Pukatzki S. Ma A. T. Revel A. T. Sturtevant D. Mekalanos J. J. (2007). Type VI secretion system translocates a phage tail spike-like protein into target cells where it cross-links actin. 104 15508–15513. 10.1073/pnas.0706532104 17873062 PMC2000545

[B104] Pukatzki S. Ma A. T. Sturtevant D. Krastins B. Sarracino D. Nelson W. C. (2006). Identification of a conserved bacterial protein secretion system in *Vibrio cholerae* using the *Dictyostelium* host model system. 103 1528–1533. 10.1073/pnas.0510322103 16432199 PMC1345711

[B105] Ramakrishnan G. (2017). Iron and Virulence in *Francisella tularensis*. 7:107 10.3389/fcimb.2017.00107 PMC5378763 28421167

[B106] Reguera G. Kolter R. (2005). Virulence and the environment: a novel role for *Vibrio cholerae* toxin-coregulated pili in biofilm formation on chitin. 187 3551–3555. 10.1128/JB.187.10.3551-3555.2005 15866944 PMC1112007

[B107] Reinhart A. A. Oglesby-Sherrouse A. G. (2016). Regulation of *Pseudomonas aeruginosa* virulence by distinct iron sources. 7:E126. 10.3390/genes7120126 27983658 PMC5192502

[B108] Reyes-Batlle M. Girbau C. Lopez-Arencibia A. Sifaoui I. Liendo A. R. Bethencourt Estrella C. J. (2017). Variation in *Campylobacter jejuni* culturability in presence of *Acanthamoeba castellanii* Neff. 183 178–181. 10.1016/j.exppara.2017.09.005 28916459

[B109] Richards A. M. Von Dwingelo J. E. Price C. T. Abu Kwaik Y. (2013). Cellular microbiology and molecular ecology of *Legionella*-amoeba interaction. 4 307–314. 10.4161/viru.24290 23535283 PMC3710333

[B110] Riquelme S. Varas M. Valenzuela C. Velozo P. Chahin N. Aguilera P. (2016). Relevant genes linked to virulence are required for *Salmonella* Typhimurium to survive intracellularly in the social amoeba *Dictyostelium discoideum*. 7:1305. 10.3389/fmicb.2016.01305 27602025 PMC4993766

[B111] Robertson P. Abdelhady H. Garduno R. A. (2014). The many forms of a pleomorphic bacterial pathogen-the developmental network of *Legionella pneumophila*. 5:670. 10.3389/fmicb.2014.00670 25566200 PMC4273665

[B112] Roig F. J. González-Candelas F. Amaro C. (2011). Domain organization and evolution of multifunctional autoprocessing repeats-in-toxin (MARTX) toxin in *Vibrio vulnificus*. 77 657–668. 10.1128/AEM.01806-10 21075892 PMC3020528

[B113] Rompikuntal P. K. Vdovikova S. Duperthuy M. Johnson T. L. Ahlund M. Lundmark R. (2015). Outer membrane vesicle-mediated export of processed PrtV protease from *Vibrio cholerae*. 10:e0134098. 10.1371/journal.pone.0134098 26222047 PMC4519245

[B114] Rossier O. Starkenburg S. R. Cianciotto N. P. (2004). *Legionella pneumophila* type II protein secretion promotes virulence in the A/J mouse model of Legionnaires’ disease pneumonia. 72 310–321. 10.1128/IAI.72.1.310-321.2004 14688110 PMC344012

[B115] Rothmeier E. Pfaffinger G. Hoffmann C. Harrison C. F. Grabmayr H. Repnik U. (2013). Activation of Ran GTPase by a *Legionella* effector promotes microtubule polymerization, pathogen vacuole motility and infection. 9:e1003598. 10.1371/journal.ppat.1003598 24068924 PMC3777869

[B116] Sack D. A. Sack R. B. Nair G. B. Siddique A. K. (2004). Cholera. 363 223–233. 10.1016/S0140-6736(03)15328-7 14738797

[B117] Samanovic M. I. Ding C. Thiele D. J. Darwin K. H. (2012). Copper in microbial pathogenesis: meddling with the metal. 11 106–115. 10.1016/j.chom.2012.01.009 22341460 PMC3285254

[B118] Sandkvist M. Michel L. O. Hough L. P. Morales V. M. Bagdasarian M. Koomey M. (1997). General secretion pathway (eps) genes required for toxin secretion and outer membrane biogenesis in *Vibrio cholerae*. 179 6994–7003. 10.1128/jb.179.22.6994-7003.1997 9371445 PMC179639

[B119] Santic M. Molmeret M. Klose K. E. Jones S. Kwaik Y. A. (2005). The *Francisella tularensis* pathogenicity island protein IglC and its regulator MglA are essential for modulating phagosome biogenesis and subsequent bacterial escape into the cytoplasm. 7 969–979. 10.1111/j.1462-5822.2005.00526.x 15953029

[B120] Santic M. Ozanic M. Semic V. Pavokovic G. Mrvcic V. Kwaik Y. A. (2011). Intra-vacuolar proliferation of *F. novicida* within *H. vermiformis*. 2:78. 10.3389/fmicb.2011.00078 21747796 PMC3128938

[B121] Sasindran S. J. Saikolappan S. Dhandayuthapani S. (2007). Methionine sulfoxide reductases and virulence of bacterial pathogens. 2 619–630. 10.2217/17460913.2.6.619 18041903

[B122] Scheid P. Schwarzenberger R. (2012). *Acanthamoeba* spp. as vehicle and reservoir of adenoviruses. 111 479–485. 10.1007/s00436-012-2828-7 22290448

[B123] Schell U. Kessler A. Hilbi H. (2014). Phosphorylation signalling through the *Legionella* quorum sensing histidine kinases LqsS and LqsT converges on the response regulator LqsR. 92 1039–1055. 10.1111/mmi.12612 24720786

[B124] Schell U. Simon S. Hilbi H. (2016a). Inflammasome recognition and regulation of the *Legionella* flagellum. 397 161–181. 10.1007/978-3-319-41171-2_8 27460809

[B125] Schell U. Simon S. Sahr T. Hager D. Albers M. F. Kessler A. (2016b). The alpha-hydroxyketone LAI-1 regulates motility, Lqs-dependent phosphorylation signalling and gene expression of *Legionella pneumophila*. 99 778–793. 10.1111/mmi.13265 26538361

[B126] Schlimme W. Marchiani M. Hanselmann K. Jenni B. (1997). Gene transfer between bacteria within digestive vacuoles of protozoa. 23 239–247. 10.1111/j.1574-6941.1997.tb00406.x

[B127] Sherr E. B. Sherr B. F. (2002). Significance of predation by protists in aquatic microbial food webs. 81 293–308. 10.1023/A:1020591307260 12448728

[B128] Shevchuk O. Pagelow D. Rasch J. Dohrmann S. Gunther G. Hoppe J. (2014). Polyketide synthase (PKS) reduces fusion of *Legionella pneumophila*-containing vacuoles with lysosomes and contributes to bacterial competitiveness during infection. 304 1169–1181. 10.1016/j.ijmm.2014.08.010 25218702

[B129] Siddiqui R. Khan N. A. (2012). Acanthamoeba is an evolutionary ancestor of macrophages: a myth or reality? 130 95–97. 10.1016/j.exppara.2011.11.005 22143089

[B130] Siddiqui R. Malik H. Sagheer M. Jung S. Y. Khan N. A. (2011). The type III secretion system is involved in *Escherichia coli* K1 interactions with *Acanthamoeba*. 128 409–413. 10.1016/j.exppara.2011.05.008 21616073

[B131] Silva A. J. Benitez J. A. (2016). *Vibrio cholerae* biofilms and cholera pathogenesis. 10:e0004330. 10.1371/journal.pntd.0004330 26845681 PMC4741415

[B132] Simon S. Schell U. Heuer N. Hager D. Albers M. F. Matthias J. (2015). Inter-kingdom signaling by the *Legionella* quorum sensing molecule LAI-1 modulates cell migration through an IQGAP1-Cdc42-ARHGEF9-dependent pathway. 11:e1005307. 10.1371/journal.ppat.1005307 26633832 PMC4669118

[B133] Simon S. Wagner M. A. Rothmeier E. Muller-Taubenberger A. Hilbi H. (2014). Icm/Dot-dependent inhibition of phagocyte migration by *Legionella* is antagonized by a translocated Ran GTPase activator. 16 977–992. 10.1111/cmi.12258 24397557

[B134] So E. C. Mattheis C. Tate E. W. Frankel G. Schroeder G. N. (2015). Creating a customized intracellular niche: subversion of host cell signaling by *Legionella* type IV secretion system effectors. 61 617–635. 10.1139/cjm-2015-0166 26059316

[B135] Steenbergen J. N. Shuman H. A. Casadevall A. (2001). *Cryptococcus neoformans* interactions with amoebae suggest an explanation for its virulence and intracellular pathogenic strategy in macrophages. 98 15245–15250. 10.1073/pnas.261418798 11742090 PMC65014

[B136] Steinberg K. M. Levin B. R. (2007). Grazing protozoa and the evolution of the *Escherichia coli* O157:H7 Shiga toxin-encoding prophage. 274 1921–1929. 10.1098/rspb.2007.0245 17535798 PMC2211389

[B137] Stolfa G. Koudelka G. B. (2012). Entry and killing of *Tetrahymena thermophila* by bacterially produced Shiga toxin. 4:e00416-12. 10.1128/mBio.00416-12 23269826 PMC3531803

[B138] Sun S. Kjelleberg S. McDougald D. (2013). Relative contributions of *Vibrio polysaccharide* and quorum sensing to the resistance of *Vibrio cholerae* to predation by heterotrophic protists. 8:e56338. 10.1371/journal.pone.0056338 23441178 PMC3575383

[B139] Suzuki M. Danilchanka O. Mekalanos J. J. (2014). *Vibrio cholerae* T3SS effector VopE modulates mitochondrial dynamics and innate immune signaling by targeting Miro GTPases. 16 581–591. 10.1016/j.chom.2014.09.015 25450857 PMC4391628

[B140] Tam V. C. Serruto D. Dziejman M. Brieher W. Mekalanos J. J. (2007). A type III secretion system in *Vibrio cholerae* translocates a formin/spire hybrid-like actin nucleator to promote intestinal colonization. 1 95–107. 10.1016/j.chom.2007.03.005 18005688

[B141] Tiaden A. Spirig T. Weber S. S. Bruggemann H. Bosshard R. Buchrieser C. (2007). The *Legionella pneumophila* response regulator LqsR promotes host cell interactions as an element of the virulence regulatory network controlled by RpoS and LetA. 9 2903–2920. 10.1111/j.1462-5822.2007.01005.x 17614967

[B142] Townsley L. Sison Mangus M. P. Mehic S. Yildiz F. H. (2016). Response of *Vibrio cholerae* to low-temperature shifts: CspV regulation of type VI secretion, biofilm formation, and association with zooplankton. 82 4441–4452. 10.1128/AEM.00807-16 27208110 PMC4959209

[B143] Trigui H. Paquet V. E. Charette S. J. Faucher S. P. (2016). Packaging of *Campylobacter jejuni* into multilamellar bodies by the ciliate *Tetrahymena pyriformis*. 82 2783–2790. 10.1128/AEM.03921-15 26921427 PMC4836424

[B144] Tyson J. Y. Pearce M. M. Vargas P. Bagchi S. Mulhern B. J. Cianciotto N. P. (2013). Multiple *Legionella pneumophila* Type II secretion substrates, including a novel protein, contribute to differential infection of the amoebae *Acanthamoeba castellanii, Hartmannella vermiformis*, and *Naegleria lovaniensis*. 81 1399–1410. 10.1128/IAI.00045-13 23429532 PMC3648003

[B145] Vaitkevicius K. Lindmark B. Ou G. Song T. Toma C. Iwanaga M. (2006). A *Vibrio cholerae* protease needed for killing of *Caenorhabditis elegans* has a role in protection from natural predator grazing. 103 9280–9285. 10.1073/pnas.0601754103 16754867 PMC1482601

[B146] Valeru S. P. Rompikuntal P. K. Ishikawa T. Vaitkevicius K. Sjöling Å Dolganov N. (2009). Role of melanin pigment in expression of *Vibrio cholerae* virulence factors. 77 935–942. 10.1128/IAI.00929-08 19103773 PMC2643646

[B147] Van der Henst C. Scrignari T. Maclachlan C. Blokesch M. (2015). An intracellular replication niche for *Vibrio cholerae* in the amoeba *Acanthamoeba castellanii*. 10 897–910. 10.1038/ismej.2015.165 26394005 PMC4705440

[B148] Vezzulli L. Colwell R. R. Pruzzo C. (2013). Ocean warming and spread of pathogenic vibrios in the aquatic environment. 65 817–825. 10.1007/s00248-012-0163-2 23280498

[B149] Vezzulli L. Pezzati E. Brettar I. Hofle M. Pruzzo C. (2015). Effects of global warming on Vibrio ecology. 3:VE-0004-2014. 10.1128/microbiolspec.VE-0004-2014 26185070

[B150] Vieira A. Ramesh A. Seddon A. M. Karlyshev A. V. (2017). CmeABC multidrug efflux pump contributes to antibiotic resistance and promotes *Campylobacter jejuni* survival and multiplication in *Acanthamoeba polyphaga*. 83:e01600-17. 10.1128/AEM.01600-17 28916560 PMC5666138

[B151] Weitere M. Bergfeld T. Rice S. A. Matz C. Kjelleberg S. (2005). Grazing resistance of *Pseudomonas aeruginosa* biofilms depends on type of protective mechanism, developmental stage and protozoan feeding mode. 7 1593–1601. 10.1111/j.1462-2920.2005.00851.x 16156732

[B152] Wheat W. H. Casali A. L. Thomas V. Spencer J. S. Lahiri R. Williams D. L. (2014). Long-term survival and virulence of *Mycobacterium leprae* in amoebal cysts. 8:e3405. 10.1371/journal.pntd.0003405 25521850 PMC4270725

[B153] Wong E. Vaaje-Kolstad G. Ghosh A. Hurtado-Guerrero R. Konarev P. V. Ibrahim A. F. M. (2012). The *Vibrio cholerae* colonization factor Gbpa possesses a modular structure that governs binding to different host surfaces. 8:e1002373. 10.1371/journal.ppat.1002373 22253590 PMC3257281

[B154] Zhou M. Guo Z. Duan Q. Hardwidge P. R. Zhu G. (2014). Escherichia coli type III secretion system 2: a new kind of T3SS? 45:32. 10.1186/1297-9716-45-32 24641581 PMC3977695

[B155] Zhu J. Miller M. B. Vance R. E. Dziejman M. Bassler B. L. Mekalanos J. J. (2002). Quorum-sensing regulators control virulence gene expression in *Vibrio cholerae*. 99 3129–3134. 10.1073/pnas.052694299 11854465 PMC122484

